# Carcinocythemia detection in peripheral blood smears: a literature review

**DOI:** 10.1515/almed-2025-0169

**Published:** 2026-03-16

**Authors:** Javier Laguna, Anna Merino

**Affiliations:** Department of Biochemistry and Molecular Genetics, 16493 CDB, Hospital Clínic de Barcelona, Barcelona, Spain; Fundació de Recerca Clínic Barcelona-Institut d’Investigacions Biomèdiques August Pi i Sunyer (IDIBAPS), Barcelona, Spain; CORE Laboratory, CDB, Hospital Clínic de Barcelona, Barcelona, Spain

**Keywords:** cancer, carcinocythemia, circulating tumor cells, peripheral blood

## Abstract

Carcinocythemia, or carcinoma cell leukemia, is a rare but striking manifestation of advanced malignancy in which circulating tumor cells (CTCs) are visible in peripheral blood (PB) smears using conventional staining. It is typically associated with advanced stage disease and poor prognosis. This review updates current knowledge on the pathophysiology, cytological features, detection methods, and clinical relevance of carcinocythemia. It explores mechanisms such as bone marrow infiltration, splenic dysfunction, and immune evasion that may facilitate tumor cell release into circulation. Morphologically, CTCs are large atypical cells often mistaken for hematologic blasts, with features that vary by tumor type. Immunocytochemistry using cytokeratins and epithelial markers (e.g. AE1/AE3, EpCAM) is crucial for confirmation. While most cases involve breast or lung cancer, other malignancies, ranging from melanoma to rhabdomyosarcoma, have also been implicated. Carcinocythemia often mimics acute leukemia and coexists with disseminated intravascular coagulation or thrombosis. To date, 95 cases have been reported, but its true prevalence may be underestimated. Recognition of this phenomenon in PB smear reviews is critical for accurate diagnosis and prognostication, especially in acutely ill or cytopenic patients. Further research is needed to elucidate its biology and clinical implications.

## Introduction

Carcinocythemia, also known as carcinoma cell leukemia, describes the striking situation in which malignant epithelial cells circulate in such abundance that they can be recognized on the peripheral blood (PB) smear review under the microscope using conventional staining, such as May Grünwald-Giemsa. These cells are known as circulating tumor cells (CTCs). The term “carcinocythemia” was introduced in 1976, when Carey et al. used it to describe two cases in which CTCs were found in PB smears obtained from two women with breast cancer [1].

CTCs were observed as early as 1869, when Thomas Ashworth described “cells identical with those of the cancer” in the blood of a man who died with disseminated cancer [2]. Since then, CTCs have been recognized as a rare event in most patients, and their detection in routine practice has generally relied on highly sensitive enrichment or molecular approaches. Their enumeration and molecular profiling have become valuable, minimally invasive biomarkers of metastatic disease and treatment response [3], 4]. Carcinocythemia, however, is defined by the direct visualization of malignant cells in the examination of the PB smear using optical microscopy and is generally associated with advanced stage disease and poor prognosis [5].

To date, the largest case series were published by Ronen et al. (seven cases) [6] and Suner et al. (22 cases) [7]. Therefore, the authors suggested that carcinocythemia is probably more frequent than previously thought and that it is now detected more often due to advances in laboratory techniques.

The aim of this review is to update current knowledge about carcinocythemia, outline the current methods for detecting CTCs in PB smear and summarize the existing literature to highlight the key features of reported cases.

## Pathophysiological considerations

When a primary tumor becomes clinically detectable, it is often estimated to contain at least 10^9^ malignant cells, as detectable by current imaging procedures such as computed tomography, magnetic resonance imaging, and positron emission tomography [8].

**Figure 1:**
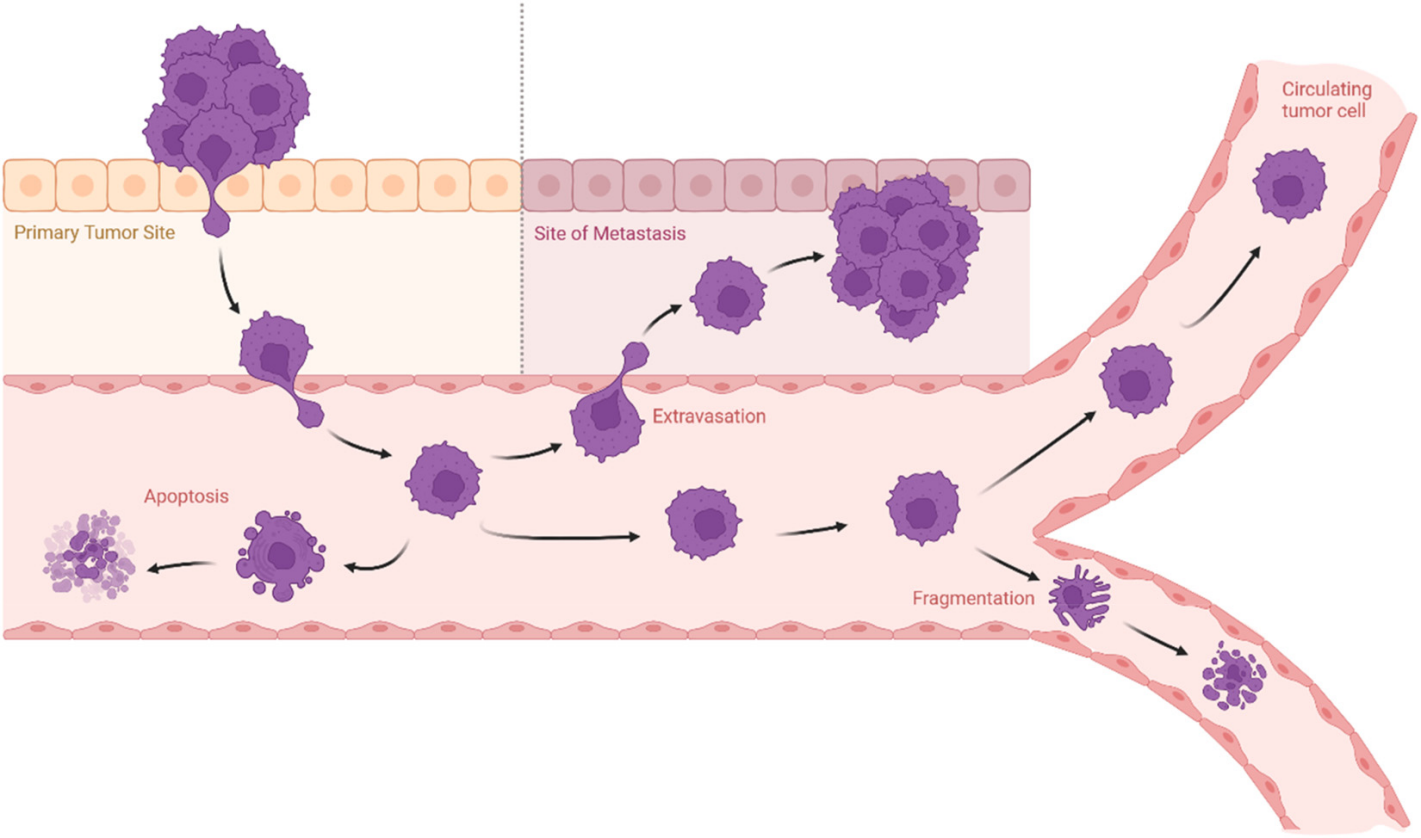
Potential outcomes for tumor cells in peripheral blood include apoptosis, fragmentation, extravasation, and continued circulation within the bloodstream. Adapted from Ref. [10]. Figure created with BioRender.

Tumor growth and metastasis are often supported by the development of new blood vessels via angiogenesis. However, alternative mechanisms such as growth along pre-existing vasculature have also been documented [9]. In any case, oxygen and nutrients are supplied to the tumor, and the tumor can also release cells that begin to circulate through the bloodstream (Figure 1). Although tumors can release millions of CTCs, their vast majority die upon entering the bloodstream, either due to apoptosis, hemodynamic stress, or fragmentation while passing through very narrow capillaries. However, some of them are able to survive and adhere to the endothelial cell lining, eventually reaching surrounding tissues and forming metastases [10]. To successfully form metastases, surviving CTCs must extravasate into distant tissues, a process facilitated by interactions between tumor cell surface molecules and endothelial adhesion receptors, as well as chemokine gradients that guide homing to specific organs [11].

Bone marrow (BM) is one of the tissues where metastasis can occur, through malignant cells invading via blood or lymphatic circulation [12]. Once infiltrated, these malignant cells may re-enter the bloodstream, contributing to the presence of CTCs in PB. In fact, most cases describe BM infiltration by malignant cells at the time of carcinocythemia [6], 7]. However, some cases occurred without BM involvement, suggesting carcinocythemia may result either from direct tumor cell release into blood or through BM entry [13], 14].

The mechanisms underlying carcinocythemia remain unclear. Some authors proposed an impaired immune response, particularly reticuloendothelial dysfunction. Ronen et al. [6] supported this hypothesis by detecting hyposplenism signs, including Howell-Jolly bodies and acanthocytes, in three of seven patients with intact spleens. This suggests that functional asplenia may hinder CTC clearance and contribute to their accumulation in PB. This finding is consistent with other reports, which also linked the presence of CTCs with either surgical splenectomy or splenic hypofunction [15], [16], [17]. However, not all patients with carcinocythemia show splenic impairment, indicating that other mechanisms, such as massive tumor burden, increased vascular invasion, BM infiltration, or chemotherapy-induced endothelial damage, may also play key roles.

## Observation of CTCs in the peripheral blood smear review

**Figure 2:**
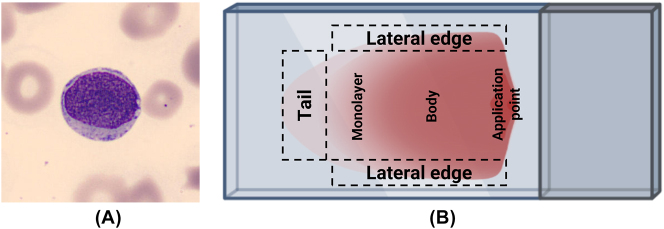
Morphological identification of circulating tumor cells and schematic representation of the peripheral blood smear regions. (A) Circulating tumor cell in peripheral blood from a patient with breast cancer. Cell image acquired with the digital analyzer CellaVision^®^ DM96 (1,000×). The presence of abundant CTCs in this patient’s peripheral blood was previously documented in Oliver-Caldes A, González-Farré B, Merino A, Rozman M. *Carcinocythaemia in an advanced stage of invasive ductal carcinoma of the breast*. Br J Haematol. 2019;185(8). (B) Schematic representation of the different regions of a peripheral blood smear. The smear is divided into the tail, lateral edges, monolayer (optimal zone for morphological analysis), body, and application point. The lateral edges and tail are areas where larger or atypical cells, including circulating tumor cells, may accumulate due to their size and should be carefully examined during microscopic evaluation. Figure created with BioRender.

Carcinocythemia can be detected in a PB smear through the identification of CTCs with distinct cytological features. In general, CTCs (Figure 2A) tend to be large or medium-sized atypical cells with a high nuclear-to-cytoplasmic (N/C) ratio, reflecting their undifferentiated or malignant nature [18], [19], [20], [21], [22], [23], [24]. Nuclear abnormalities include pleomorphic, hyperchromatic, or irregular nuclei, often with prominent nucleoli and chromatin patterns ranging from fine and reticular to coarse and clumped [24], [25], [26], [27], [28]. The cytoplasm is usually basophilic, often vacuolated or with poorly defined borders [29], [30], [31], [32]. CTCs are often non-cohesive but may appear in small clusters [6], 14], 29].

In some cases, their morphology mimics hematopoietic cells such as lymphoblasts, myeloblasts, or plasma cells, leading to misclassification as hematologic malignancies. Given the potential for misinterpretation, Table 1 summarizes key cytomorphologic features and recommended studies to distinguish CTCs from common hematologic mimickers on PB smears. Some of the CTCs show a very characteristic appearance, such as signet ring cell morphology [33], 34], multinucleated giant cells [26] or melanin pigment [25]. Despite its variability, the recognition of the described morphological patterns should lead to consideration of the presence of carcinocythemia in patients with advanced malignancy. In subsequent sections, we explore the specific morphological features of CTCs according to tumor type, which can offer additional diagnostic value in identifying the origin and nature of the underlying malignancy.

**Table 1:** Key cytomorphologic features and ancillary studies to distinguish circulating tumor cells from common hematologic mimickers on peripheral blood smears.

Feature	Circulating tumor cells	Myeloblasts	Atypical promyelocytes	Lymphoblasts	Plasma cells	Abnormal lymphocytes
Typical distribution on smear	Often enriched at feathered edge/tail; may appear in clusters	Usually evenly distributed; single cells	Even distribution; single cells	Even distribution; single cells	Even distribution; single cells	Even distribution; single cells (rare clustering)
Cell size/pleomorphism	Often large with marked pleomorphism	Medium-large, more uniform	Large, relatively uniform	Small–medium, relatively uniform	Medium; eccentric nucleus gives “clock-face” impression	Variable, often relatively uniform within a case
Nuclear contour	Frequently irregular, angulated; nuclear membrane thickening may be seen	Round/oval; smoother contours	Round/oval	Round/oval	Eccentric, round/oval	Round to irregular depending on subtype
Chromatin	Ranging from fine and reticular to coarse and clumped	Fine	Slightly more condensed than blasts	Fine	Dense heterochromatin clumped at the nuclear periphery	Typically, more clumped than blasts; may be mature-appearing
Nucleoli	Prominent (often multiple), but variable by tumor type	Prominent	May be visible but often less prominent than blasts	Prominent, but may be inconspicuous	Usually, inconspicuous	Variable; often inconspicuous in mature lymphomas
Cytoplasm	Moderate to abundant; may show sharp borders, vacuoles; mucin may create signet-ring appearance; melanin pigment in melanoma	Scant–moderate; basophilic	Moderate; heavy azurophilic granules	Scant; basophilic	Deep basophilia with perinuclear clear area	Variable; often scant–moderate
Vacuolization	Common (especially, adenocarcinoma); may be large	Uncommon (may occur)	Uncommon	Uncommon	Uncommon	Uncommon
Granules/Auer rods	Absent	May show rare granules; Auer rods can be present in AML	Prominent primary (azurophilic) granules; needle- or shard-like cytoplasmic inclusions (“splinters”) may be seen	Absent	Absent	Absent
Recommended ancillary studies (when needed)	Immunocytochemistry on smear/cell block: pan-cytokeratin (AE1/AE3), CK8/18, EMA, EpCAM (tumor-type markers as appropriate); CD45 negative.	Flow cytometry: CD34, CD117, MPO; cytogenetics/molecular	Flow cytometry: CD33, MPO, APL profile; *PML*-*RARA* testing	Flow cytometry: TdT, CD34, lineage markers	Flow cytometry: CD38, CD138, light-chain restriction	Flow cytometry: B/T markers; clonality studies

AE1/AE3, pan-cytokeratin antibody cocktail (cytokeratin AE1 + AE3); AML, acute myeloid leukemia; APL, acute promyelocytic leukemia; CD, cluster of differentiation; CK, cytokeratin; EMA, epithelial membrane antigen; EpCAM, epithelial cell adhesion molecule; MPO, myeloperoxidase; TdT, terminal deoxynucleotidyl transferase.

CTCs are generally found at the tail and the lateral edges of the smear due to their large size (Figure 2B) [35], 36]. The tail, with wide cell spacing, is often the best place to observe platelet aggregates, parasites such as microfilariae, and large atypical cells [37]. The same applies to the lateral edges of the smear, which should also be carefully examined for similar findings.

Additionally, to improve the detection of these cells, an enrichment process can sometimes be employed, such as separation by density gradient (e.g., Ficoll gradient), producing a buffy coat containing all mononuclear cells, including CTCs if present [38]. This layer can then be microscopically examined for carcinocythemia. However, it should be noted that pre-analytical processing and enrichment procedures (including centrifugation and concentration steps) may introduce morphological artifacts or alter cellular appearance compared with a standard PB smear.

## Immunocytochemical stains

Immunocytochemical staining techniques can help determine cell lineage. The most common markers are those present in epithelial cells and absent in hematopoietic cells, such as cytokeratins (CK) and epithelial cell adhesion molecules (EpCAM), widely used since most tumor cells express them [8], 39].

CK are intermediate filament proteins forming part of the cytoskeleton in epithelial cells. Their expression varies by tissue, enabling identification of the primary origin of metastases [40]. One of the most widely used stains is AE1/AE3, a cocktail of two different monoclonal antibody clones (AE1 and AE3) that together serve as a broad-spectrum cytokeratin marker. The AE1 antibody targets cytokeratins 10, 13 to 16, and 19, while AE3 detects cytokeratins 1 to 8 [41]. This broad reactivity makes AE1/AE3 useful for identifying epithelial tumor cells across cancer types. CK19 is another common marker, with strong and consistent expression in CTCs from breast cancer [42], but also positive in lung or gastrointestinal cancer [43]. Another antibody is CAM 5.2, which is specific for CK8 (and, to a lesser extent, for CK7) [44].

EpCAM are transmembrane glycoproteins involved in adhesion, normally expressed on epithelial cells and overexpressed in many carcinomas. Antibodies such as Ber-EP4 [45] target epitopes present on epithelial but absent in mesothelial and hematopoietic cells [46], making it particularly useful for distinguishing malignant epithelial cells.

Besides epithelial markers, mesenchymal and tumor-specific markers can be used, such as HER2 or ER in breast cancer, and Melan-A or HMB-45 in melanoma. Additionally, it is important to note that CTCs show negativity for CD45, which distinguishes them from cells of hematopoietic origin [39].

## Clinical utility

Carcinocythemia is more than an academic curiosity: it is a red flag for terminal-stage disease and can confound diagnosis by mimicking hematologic malignancies, most often acute leukemia [47], 48],but occasionally lymphoma or plasma cell leukemia [49], [50], [51]. Therefore, the presence of CTCs in PB may present diagnostic challenges for laboratory experts, as these cells can morphologically resemble abnormal hematopoietic cells, potentially leading to misdiagnosis. Recognizing the entity and understanding its biological implications remain essential for accurate diagnosis, appropriate supportive care, and realistic prognostication.

Quantifying the number of CTCs is important, as the clinical validity of CTCs has been well established across multiple cancer types and disease stages, including both localized and metastatic settings [52]. Regarding breast cancer, analyses in early-stage disease have demonstrated that the presence of at least one CTC is an independent prognostic factor for both disease-free survival and overall survival [53]. Similarly, in a large multicenter study, metastatic breast cancer patients with ≥5 CTCs per 7.5 mL of blood, as measured by the CellSearch System, had significantly shorter progression-free and overall survival compared to those with lower counts [54]. Similar prognostic significance has been demonstrated in patients with gastrointestinal [55], [56], [57], lung [58], 59], melanoma [60], ovarian [61], 62], and urothelial cancer [63], [64], [65], among others.

All studies on the clinical utility of CTCs rely on the CTC count by detection methods such as molecular techniques or flow cytometry, rather than the observation of CTCs in PB. To date, no studies have assessed the correlation between the presence of morphologically visible CTCs and outcomes such as disease-free or overall survival. However, documented cases consistently indicate that patients often die shortly after CTCs become visible in PB [21], 27], 31], 66], 67]. The survival ranged from one day to 10 months. These findings suggest that the CTCs detection in PB may serve as a biomarker of advanced-stage disease and very poor short-term prognosis, underscoring the need for further prospective research.

While most current clinical applications of CTCs depend on specialized detection systems and quantification, the rare but striking presence of CTCs in PB could function as a valuable and accessible clinical tool. PB smear review may offer a rapid and low-cost screening strategy in patients with cancer and unexplained cytopenias, signs of disseminated intravascular coagulation (DIC), or suspected BM failure. Additionally, in patients with no confirmed diagnosis of cancer but presenting with atypical circulating cells, severe anemia, or thrombocytopenia of unknown origin, the presence of epithelial-appearing cells in PB may provide the first clue toward a solid tumor diagnosis [20], 33], 49].

## Advantages and limitations

**Figure 3:**
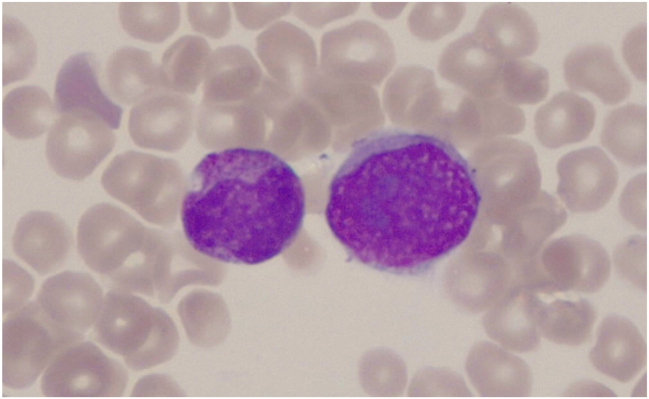
Circulating tumor cell (CTC, right) in peripheral blood, located adjacent to an immature granulocyte (left). The CTC displays a markedly high nucleus-to-cytoplasm ratio and several prominent nucleoli. This CTC was observed in a patient with gastric cancer at the time of diagnosis and was reported in: Rakislova N, Cuatrecasas M, Molina A, Rodrigo M, Moreira L, Laguna J, et al. Signet ring cell carcinocythaemia in an advanced gastric carcinoma. Int J Lab Hematol. 2020 Oct;42(5):e231–e233. Image acquired with Leica DM500 microscope (1,000×).

Observing intact CTCs in the PB smear examination can provide valuable information about the type of disease. For example, in the case reported by us [33], CTCs were identified in PB at the time of diagnosis in a patient with gastric cancer (Figure 3). Some of the observed cells were signet ring cells, characterized by a large vacuole and most frequently associated with gastric cancer, though they can also be found, less commonly, in prostate, colon, and ovarian cancers. Beyond morphology, PB smear review is a widely accessible and low-cost tool that can be performed quickly in most laboratories and may raise early suspicion of carcinocythemia while more specific confirmatory tests are being arranged.

However, CTCs are usually present in very low numbers and are extremely fragile [68], making them difficult to detect and increasing the risk of false-negative results. Pre-analytical and analytical factors may further compromise evaluation, including delays between blood collection and smear preparation/staining, suboptimal smear quality, and cell degeneration during handling. In addition, CTC morphology may overlap with hematologic mimickers, and therefore, when suspicious cells are identified, confirmation with ancillary studies (e.g., immunocytochemistry for epithelial markers and lack of CD45 expression, when feasible) is essential to support an accurate interpretation.

## Published cases in the scientific literature

We performed a structured literature search in PubMed/MEDLINE, Scopus, ResearchGate, and Web of Science from the earliest records available to September 2025 using combinations of the following terms: “carcinocythemia”, “carcinocythaemia”, “carcinoma cell leukemia”, “carcinoma cell leukaemia”, “circulating tumor cells”, and “peripheral blood smear”, as well as “peripheral blood film”. Reference lists of relevant articles and available case series were also screened to identify additional reports. We included publications reporting morphologically visible circulating tumor cells on conventional PB smears, and excluded studies focused exclusively on enrichment-based platforms without smear documentation.

**Figure 4:**
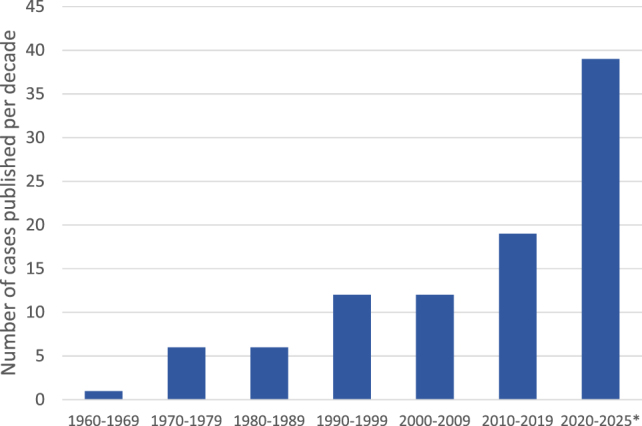
Number of reported carcinocythemia cases by decade. The graph shows the temporal distribution of published cases, with a notable increase in reports, particularly during the ongoing decade (*).

Since 1960, 95 cases of carcinocythemia have been reported (Supplementary Table 1). The number of reported cases has increased in recent years (Figure 4), with 39 documented cases in the current decade. While single case reports dominated for decades, larger series are emerging. Ronen et al. reported seven cases at one institution over three years, suggesting the phenomenon may be under-recognized [6]. Most recently, Suner et al. collected 22 cases from 15 French centers, providing the largest multi-center overview to date [7].

Most reported cases occurred in women (66 out of 95 cases, 69 %). The average age was 53 years (range: 12–80 years). The youngest patients had Ewing sarcoma (12 years) [22] and rhabdomyosarcoma (13 years) [69], while the oldest patients (80 years) had transitional cell carcinoma [14] and breast cancer [32].

**Figure 5:**
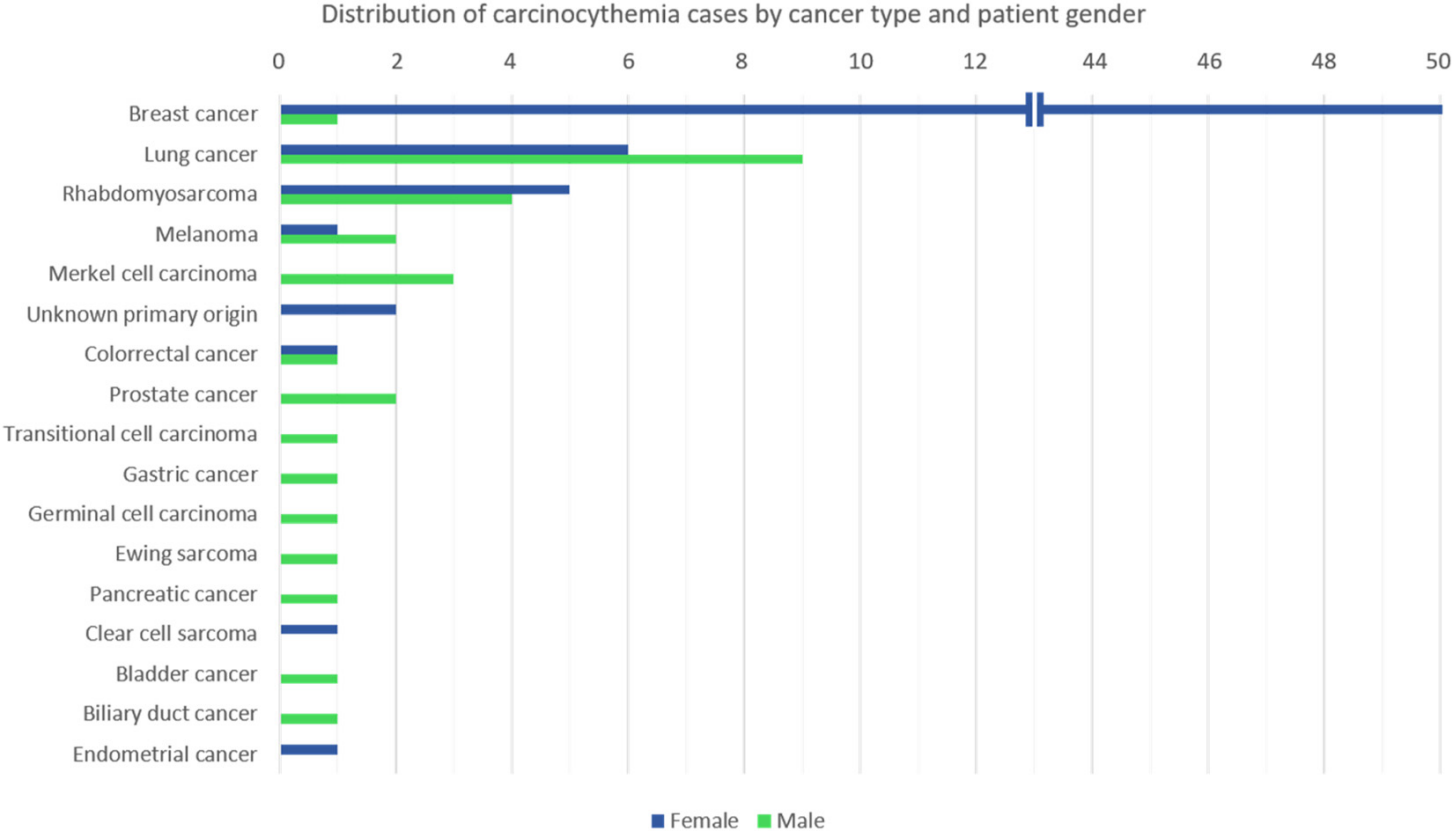
Distribution of carcinocythemia cases by cancer type and patient gender. The chart illustrates the frequency of reported carcinocythemia cases across different tumor origins, highlighting a predominance in breast and lung cancers. The gender distribution reflects a higher incidence in women, primarily due to the high number of breast cancer-associated cases.

Breast carcinoma is the most common associated malignancy (Figure 5), followed by lung cancer. Most patients had a prior diagnosis of cancer, although some cases have been described at the time of initial diagnosis [33], [34], [35, 50], 70], 71].

Table 2 summarizes the key outcomes reported across published cases, using available-case denominators where variables were explicitly stated. Concerning BM involvement, all except three of the reported cases in the literature [13], 14], 72] showed infiltration of the BM by malignant cells. Coagulation complications were observed in 26/55 cases (47 %), including DIC in 7/55 (13 %). Among fatal cases with quantifiable follow-up, the median time from CTC detection to death was 15 days (range: 1–304 days).

**Table 2:** Summary of key outcomes reported in published cases of carcinocythemia with morphologically identifiable circulating tumor cells on peripheral blood smears.

Outcome	No./total (%)
Bone marrow infiltration	59/62 (95 %)
Any coagulation complication	26/55 (47 %)
Disseminated intravascular coagulation	7/55 (13 %)
Death reported during follow-up	49/70 (70 %)

Laboratory findings show wide variability, although most patients presented with anemia and thrombocytopenia. The proportion of CTCs in the PB smear can range from 1 to 80 % of the total white blood cell count, although low percentages are most common. Of the 95 cases, 56 included explicit information on the proportion of malignant cells. Among these, 18 patients had less than 10 % abnormal cells, while in another 15 cases only a single cluster, a few, or isolated abnormal cells were described. Additionally, thrombosis and DIC are frequently associated with carcinocythemia. This is thought to result from the combination of tumor-induced hypercoagulability, endothelial activation, and extensive marrow involvement. CTCs can express procoagulant molecules such as tissue factor and cancer procoagulant, which directly activate the coagulation cascade and promote a systemic hypercoagulable state [73]. Moreover, most cases of carcinocythemia occur in the context of widespread BM infiltration, which disrupts hematopoiesis and triggers cytokine release that contributes to endothelial dysfunction [20]. The high burden of circulating malignant cells can also cause vascular injury and platelet activation, further favoring thrombus formation [25]. Inflammatory cytokines like IL-1, IL-6, and TNF-α, commonly elevated in advanced cancer, can further enhance the prothrombotic microenvironment and contribute to DIC [74]. Several case reports document the coexistence of carcinocythemia with thrombosis or overt DIC, suggesting that these phenomena are part of the same pathophysiological spectrum in terminal-stage malignancy [27], 31], 49].

## Breast cancer

Breast cancer is the most frequently reported malignancy associated with carcinocythemia, accounting for 50 documented cases (53 %) [1], [5], [6], [7, 15], [19], [20], [21, 23], 24], 27], 28], 31], 32], 35], 36], [48], [49], [50], [51, 66], 70], 71], [75], [76], [77], [78], [79], [80], [81], [82], [83]. It usually appears in advanced metastatic disease, often terminal, and predominantly in women, although at least one male case has been reported [48].

**Figure 6:**
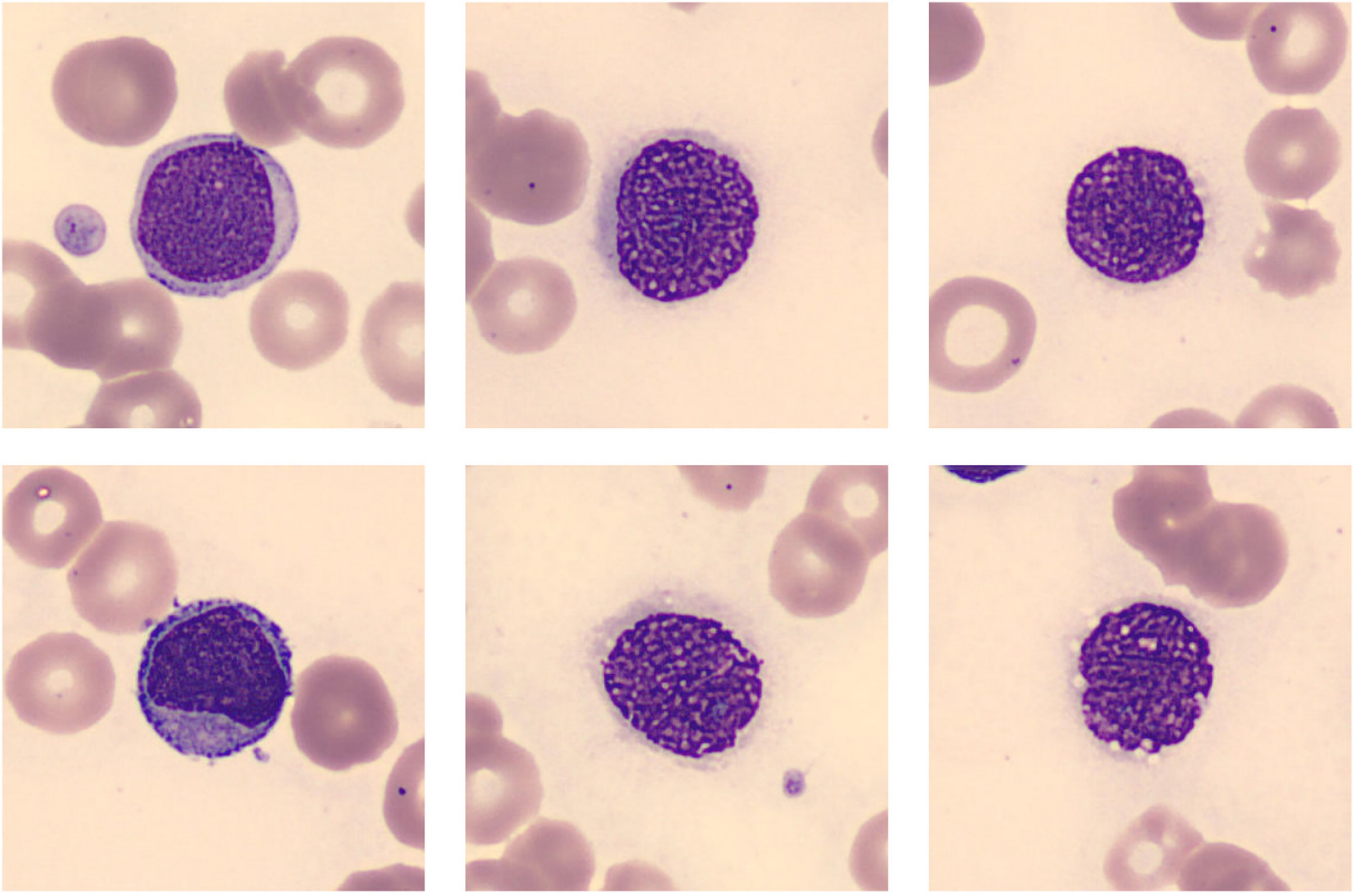
Circulating tumor cells observed in peripheral blood from the patient with breast cancer previously reported by Oliver-Caldes A, González-Farré B, Merino A, Rozman M. Carcinocythaemia in an advanced stage of invasive ductal carcinoma of the breast. Br J Haematol. 2019;185(8). Both intact cells and disrupted cells with only nuclei visible can be seen. Images acquired with CellaVision^®^ DM96 (1,000×).

Morphologically, the CTCs are large, pleomorphic cells with high N/C ratios, prominent nucleoli, and basophilic or vacuolated cytoplasm. In some instances, due to the fragility of CTCs, only nuclei may be visible (Figure 6). CTCs are often arranged in clusters and may mimic hematologic malignancies, such as acute leukemia or Burkitt lymphoma [27], 31], 66], 76]. Immunocytochemical staining is essential for confirming their epithelial origin, with cytokeratins (e.g. CK AE1/AE3) and epithelial membrane antigen commonly used. Some CTCs have shown a signet ring-like morphology, especially in cases with abundant vacuolization [15], 66].

Clinically, most patients presented with cytopenias, DIC, or thrombotic microangiopathy, and the detection of carcinocythemia often preceded rapid clinical decline. Prognosis is universally poor. In nearly all cases, patients died within days to a few weeks of CTC detection, despite supportive or palliative treatment. Some exceptions have been reported – such as a case responding briefly to trastuzumab – but overall survival remains extremely limited once carcinocythemia develops [20].

This group of cases underscores the diagnostic importance of the PB smear review in advanced breast cancer and demonstrates that the presence of circulating CTCs is a late and ominous sign, often associated with BM involvement, coagulopathy and impending death.

## Lung cancer

Lung cancer is the second most frequently associated primary malignancy in reported cases of carcinocythemia, following breast cancer, with 15 documented patients to date, representing approximately 30 % of published cases in the available literature [6], 7], 13], 67], [84], [85], [86], [87], [88], [89], [90]. The majority of these cases involved small cell lung carcinoma (SCLC), which emerged as the predominant histological subtype among patients with lung cancer-associated carcinocythemia [13], [84], [85], [86], [87], [88], [89], [90]. However, a few cases of non-small cell carcinoma have also been reported [6], 67].

A common morphological pattern described in these SCLC cases includes medium-to-large CTCs with a high N/C ratio, irregular nuclei, scant cytoplasm, and frequently clustered, especially at the edge of the PB smear. In the few reported NSCLC cases with detailed morphology, the CTCs are described as atypical, large single cells with folded/irregular nuclei, prominent nucleoli, and basophilic cytoplasm with abundant vacuolization.

Several reports highlight that carcinocythemia in lung cancer can sometimes be the first indication of malignancy. For instance, in the Suner et al. cohort, four of the six patients diagnosed with carcinocythemia as an initial finding were later found to have lung cancer [7]. This underlines the diagnostic importance of carefully examining blood smears, especially in acutely ill patients.

Clinical presentations included severe cytopenias, elevated lactate dehydrogenase, and DIC. In most cases, patients deteriorated rapidly after the appearance of CTCs in the PB examination, with survival ranging from hours to a few weeks following diagnosis. For example, a 60-year-old man with Stage IV adenocarcinoma reported by Samal et al. died within hours of CTCs detection, despite prior chemotherapy. This case was particularly remarkable due to the rare simultaneous presence of microfilaria in the PB [67].

## Rhabdomyosarcoma

Carcinocythemia in rhabdomyosarcoma is rare and generally indicates advanced, disseminated disease. To date, nine cases have been described, often involving children or young adults (ages 13–26) who presented with severe anemia, thrombocytopenia, and tumor cells detection in PB [29], 30], 47], 69], 72], 91], 92]. These circulating cells typically mimic hematopoietic blasts: they show high N/C ratio, fine chromatin, inconspicuous nucleoli, and can appear round, oval, or spindle-shaped. Clumping, syncytial clusters, or rosette-like arrangements have also been observed in BM or smears, and BM infiltration is almost universal at this stage.

These cases are frequently misdiagnosed initially as acute leukemia, highlighting the importance of using cytochemistry, immunocytochemistry, and molecular testing to differentiate rhabdomyosarcoma from true hematologic malignancies [30], 47]. Collectively, these reports emphasize the need for awareness of this rare manifestation, especially when blast-like cells are detected in young patients with unexplained cytopenias and masses suspicious for rhabdomyosarcoma.

## Melanoma

Carcinocythemia in malignant melanoma, sometimes referred to as melanocythemia [93], is a rare and aggressive manifestation of disseminated disease, with only a few published cases to date. Trefzer et al. described a striking example of amelanotic melanoma presenting with a leukemic picture, where PB showed 30–40 % large pleomorphic cells with abundant cytoplasm, eccentric nuclei, and absence of melanin pigment. These cells mimicked blasts but were confirmed as melanoma by positive immunostaining for S100, vimentin, and HMB-45, and by their lack of hematologic markers [93]. In contrast, the case reported by Tran et al. featured melanotic melanoma cells in both PB and BM, some of which contained visible melanin granules, offering a key morphological clue [25]. In both cases, BM was extensively infiltrated, and the patients deteriorated rapidly, dying within days or weeks of diagnosis, highlighting the poor prognosis associated with this presentation.

While melanotic melanoma may be more readily suspected due to its pigmented appearance, amelanotic forms can easily be misdiagnosed as hematologic malignancies without specific immunohistochemistry. Gallivan et al. had earlier described a similar case of carcinocythemia in metastatic melanoma, noting the diagnostic difficulty when CTCs resemble immature hematopoietic precursors [14]. Overall, the presence of melanoma cells in PB represents an advanced disease state with limited therapeutic options, and recognition of both morphological and immunophenotypic features is essential for accurate diagnosis.

## Merkel cell carcinoma

Merkel cell carcinoma (MCC) is a rare and aggressive neuroendocrine skin tumor that can exceptionally present with carcinocythemia. To date, only three cases have been reported: two by Tam et al. [94] and one by Hartley et al. [95]. In all cases, the patients had advanced disease with extensive BM involvement and varying degrees of immunosuppression. The CTCs observed in blood smears exhibited non-hematopoietic morphology, including deeply basophilic cytoplasm, oval nuclei with open chromatin, and multiple prominent nucleoli. These cells were identified as MCC by immunohistochemistry – demonstrating CD56 and cytokeratin positivity – and by flow cytometry, revealing CD56^+^/CD45^−^ phenotypes without myeloid or lymphoid markers.

Despite slight differences in clinical background, all three cases shared features of aggressive disease, rapid progression, and poor outcome. Notably, two of the patients died within weeks of diagnosis, and one was lost to follow-up shortly after discharge, reinforcing the grave prognosis associated with circulating MCC cells.

## Colorectal cancer

Carcinocythemia in colorectal cancer is extremely rare, with only two published cases to date: one by Misawa et al. [96] and another by van Bunderen et al. [97]. Both patients had advanced mucin-producing tumors, specifically, signet ring cell carcinoma (SRCC) and mucinous adenocarcinoma of the sigmoid colon, respectively. In both cases, atypical CTCs with signet ring morphology were identified in the PB smear and confirmed by immunocytochemistry using epithelial markers such as CAM 5.2 and mucin stains. BM infiltration was present in both patients, suggesting that the marrow may act as a reservoir from which tumor cells are released into the bloodstream.

Clinically, both patients developed DIC and had poor prognoses. The patient described by Misawa et al. died within three days of diagnosis without receiving treatment, whereas the case reported by van Bunderen et al. achieved temporary disease stabilization following chemotherapy, surviving nearly eight months before succumbing to disease progression. Despite the therapeutic response in one case, the presence of circulating signet ring cells remains a marker of aggressive disease and systemic dissemination.

## Prostate cancer

Carcinocythemia in prostate cancer is an exceptionally rare phenomenon, with only two cases reported to date: one described by Ronen et al. [6] and the other by Jain and Wang [17]. Both cases involved elderly male patients with advanced, metastatic prostate carcinoma and presented with anemia and thrombocytopenia. In both reports, PB smears revealed clusters of large atypical cells at the tail, with prominent nucleoli, irregular nuclear contours, and moderately abundant basophilic cytoplasm, features that helped distinguish them from hematopoietic cells. In Jain and Wang’s case, the diagnosis was confirmed by pancytokeratin and prostate-specific antigen (PSA) positivity on BM biopsy, while the BM examination was not performed in the patient reported by Ronen et al. Despite some initial clinical improvement in the patient described by Jain and Wang following radiation and systemic anti-androgen therapy, the presence of carcinocythemia reflected a highly disseminated disease. The patient reported by Ronen et al. died just two months after the diagnosis of carcinocythemia, reinforcing its association with poor prognosis and short-term survival.

## Transitional cell carcinoma

Carcinocythemia has been exceptionally reported in transitional cell carcinoma (TCC), with only one documented case in the literature to date. In 1984, Gallivan and Lokich described an 80-year-old male patient with metastatic TCC of the ureteropelvic junction who developed carcinocythemia in the final stage of disease [14]. The PB smear review revealed numerous clusters of tumor cells with large hyperchromatic nuclei and a high N/C ratio, consistent with non-hematologic malignancy. Interestingly, despite the presence of CTCs, BM and spleen were free of metastasis.

The patient’s clinical course was marked by pancytopenia and DIC, and he ultimately died eight weeks after diagnosis. This case underscores the aggressive nature of advanced TCC and the potential for epithelial tumor cells to reach the PB even in the absence of BM involvement.

## Gastric cancer

Carcinocythemia in gastric cancer is extremely rare, with only one documented case to date, reported by our group [33]. The patient, a 32-year-old man, presented with advanced diffuse-type gastric carcinoma and systemic dissemination at diagnosis. Remarkably, carcinocythemia was observed even before diagnosing cancer. The PB smear review revealed large atypical epithelial cells with prominent nucleoli and high N/C ratios, alongside intermediate to large signet ring cells, a distinctive morphological feature of poorly cohesive gastric carcinoma. These findings were confirmed by immunocytochemistry using Ber-EP4 and AE1/AE3, supporting the epithelial origin of the circulating cells.

As in other reports of carcinocythemia, the patient exhibited severe cytopenias and BM infiltration. A germline *CDH1* mutation, associated with poor prognosis in hereditary diffuse gastric cancer [98] may have facilitated early dissemination. Although only a single case has been reported, it highlights the aggressive behavior of signet ring cell gastric carcinoma and the diagnostic value of blood smear review, especially when combined with immunocytochemistry. Detection of such cells generally indicates widespread disease and is associated with rapid deterioration and poor prognosis.

## Germinal cell carcinoma

Carcinocythemia has been exceptionally reported in malignant germ cell tumors, with a single documented case to date. Irie et al. described a 35-year-old man diagnosed post-mortem with an extragonadal germ cell tumor of the anterior mediastinum, histologically consistent with an immature teratoma containing embryonal carcinoma-like components and Schiller-Duval body-like structures [99]. During his clinical course, numerous atypical cells with large, hyperchromatic, bizarre nuclei and scant cytoplasm were found in the PB smear, closely resembling hematologic malignancies. However, immunohistochemical and cytochemical analysis ruled out hematopoietic origin. According to the authors, this was the first report of carcinocythemia in a malignant germ cell tumor, and they hypothesized that the extensive BM involvement might have facilitated the release of tumor cells into circulation, mimicking acute leukemia.

## Ewing sarcoma

Carcinocythemia associated with Ewing sarcoma is an extremely rare event. In 2023, Zhang et al. [22] reported the case of a 12-year-old boy with metastatic Ewing sarcoma who developed concurrent carcinocythemia, along with chest wall and right knee joint involvement. The patient had been undergoing maintenance chemotherapy and initially responded well to treatment. However, he later presented with joint pain and laboratory abnormalities including leukocytosis and severe thrombocytopenia. Remarkably, PB analysis revealed large abnormal cells with a high N/C ratio, round nuclei, and fine chromatin, resembling lymphoblast-like morphology. Using the buffy coat method, clusters of malignant cells were also observed at the feathered edge of the smear, confirming the presence of CTCs.

This case illustrates several key features of carcinocythemia in pediatric Ewing sarcoma: its occurrence even in patients previously responding to therapy, the potential for mimicking hematologic malignancies morphologically, and the importance of using both central smear review and buffy coat concentration for accurate detection.

## Pancreatic cancer

Carcinocythemia has been reported only once in association with pancreatic cancer. Usnarska-Zubkiewicz et al. described a 33-year-old man diagnosed with pleomorphic giant cell carcinoma, a rare and aggressive variant of pancreatic ductal adenocarcinoma [26]. The patient presented with severe anemia, thrombocytopenia, and leukocytosis, accompanied by atypical immature cells in PB and BM. These circulating cells, morphologically blast-like with pleomorphic and multinucleated osteoclast-like features, mimicked acute leukemia. Immunophenotyping confirmed the epithelial origin of these cells (negative for CD45 and positive for vimentin and epithelial membrane antigen). Despite palliative surgery, the patient died just three days after admission, underscoring the rapid and fatal course of carcinocythemia in this context.

## Clear cell sarcoma

Clear cell sarcoma (CCS), a rare and aggressive soft tissue malignancy, has only exceptionally been associated with carcinocythemia. The first reported case involved a middle-aged woman who developed widespread metastases and serous involvement of the pleura and pericardium [100]. Two months after initial diagnosis, the PB smear review revealed clusters of malignant cells, some of which contained pigment particles. The cells exhibited eccentric round nuclei, prominent nucleoli, abundant cytoplasm with microvacuoles, and resembled plasmablastic morphology, although immunohistochemistry was negative for plasma cell markers (CD138, CD38). Tumor cells expressed Melan-A, S-100, and HMB-45, consistent with CCS, and notably mimicking malignant melanoma.

In addition, cytology of pleural and pericardial effusions revealed tumor clusters and mitoses, reflecting the aggressive biology of CCS. This aggressive behavior is consistent with the known biology of CCS, which typically affects young adults and often presents with melanocytic markers. Molecular testing, particularly detection of the *EWSR1::ATF1* fusion, is crucial to distinguish CCS from melanoma [101]. This case illustrates how CCS can present with a leukemia-like picture in blood, and that meticulous morphological assessment of both blood and effusion samples can offer critical diagnostic clues in metastatic disease.

## Bladder cancer

A single case has linked carcinocythemia to bladder cancer, specifically a rare signet ring cell carcinoma (SRCC) subtype [34]. The patient, a 65-year-old man, initially presented with skeletal metastases, profound cytopenias, and numerous CTCs in PB showing classic signet ring features. The primary tumor site remained undetected during life, and a definitive diagnosis was made only post-mortem, when autopsy revealed diffuse infiltration of the bladder wall by SRCC. This case is notable not only for being the first report of carcinocythemia secondary to primary bladder SRCC, but also for highlighting the diagnostic difficulty of cancer of unknown primary (CUP) in the context of rare histological subtypes and unusual presentations.

## Biliary duct cancer

Carcinocythemia has also been reported in a 63-year-old man with biliary tract cancer [7]. The multicenter study by Suner et al. described the CTCs as highly atypical – ranging from medium to very large in size, often multinucleated, with reticulated to coarse chromatin, prominent nucleoli, and abundant basophilic cytoplasm. These cytological features reflect the aggressive biology of biliary duct carcinoma and its potential for widespread dissemination.

## Endometrial cancer

A case of carcinocythemia was identified in a 79-year-old woman with endometrial cancer [7]. While individual clinical details were limited, the CTCs observed in PB were described as large cells with round nuclei, dispersed chromatin, prominent nucleoli, and deeply basophilic cytoplasm. These features highlight the potential of endometrial carcinoma to disseminate systemically and present with blood findings that may resemble acute leukemia or other hematologic disorders.

## Cancer of unknown primary origin

Although carcinocythemia is typically associated with advanced-stage epithelial tumors of known origin, there are rare instances in which the primary site remains unidentified, even after extensive diagnostic workup. In 1996, Nasr et al. reported a 55-year-old woman who initially presented with asthenia and mild hyperlymphocytosis. The PB smear review revealed 10 % tumor cells, morphologically resembling lymphocytes but lacking CD45 expression. Immunocytochemistry was strongly positive for epithelial markers associated with breast cancer. Although BM biopsy revealed extensive infiltration by adenocarcinoma cells, imaging failed to identify a clear primary tumor. The patient responded to chemotherapy with resolution of splenomegaly and peripheral tumor cells, and notably survived for more than 20 months on maintenance hormonal therapy (a rare case of prolonged survival following carcinocythemia) [16]. More recently, Ronen et al. presented a case involving a 65-year-old woman with widespread metastases, severe cytopenias, and CTC clusters in PB, yet with no identifiable primary site even after extensive radiological and immunohistochemical studies [6]. Despite aggressive disease and palliative care, the patient deteriorated and died shortly after diagnosis.

## Conclusions

Carcinocythemia remains an exceptionally rare but clinically significant phenomenon, often heralding terminal-stage disease and complicating routine hematologic diagnostics. Although the total number of reported cases is still limited, their consistent association with severe cytopenias, BM infiltration, and rapid clinical decline highlights its prognostic relevance. Despite advances in molecular CTC detection and enrichment technologies, the presence of morphologically visible CTCs in PB smear remains an accessible yet underexplored marker of advanced malignancy.

This review has limitations inherent to the available evidence: most data come from isolated case reports, often published only when the phenomenon is striking, introducing publication bias and limiting generalizability. Few studies systematically document laboratory values, follow-up, or standardized smear examination techniques. These gaps emphasize the need for prospective multicenter studies and methodological standardization to clarify the biological and clinical implications of carcinocythemia.

For now, careful PB smear review, combined with confirmatory immunohistochemistry or molecular testing when needed, remains essential to avoid misdiagnosis and ensure appropriate supportive care and realistic prognostication.

## Supplementary Material

Supplementary Material
