# Supplementary material for: Carcinocythemia detection in peripheral blood smears: a literature review
Source: Adv Lab Med. 2026 Mar 16;7(2):90–104. doi: 10.1515/almed-2025-0169 (PMC13169457; doi:10.1515/almed-2025-0169)
Supplement: Supplementary file 1 — Supplementary Material [file j_almed-2025-0169_suppl_001.pdf]

Supplemental Table 1. Published cases of carcinocythemia with morphologically identifiable circulating tumor cells in peripheral blood smears: clinical characteristics, laboratory findings, morphological features, and outcomes.

Year of publication	Ref	Authors	Age	Gender	Cancer type	CTC features	Leucocytes (x10^9/L)	Hemoglobin (g/L)	Platelets (x10^9/L)	BM infiltration	Misdiagnosing with malignant hematologic diseases?	Which disease?	Personal history of cancer	Coagulation complications?	Which coagulation complications?	Death	Time to death
1960	[13]	Finkel and Tishkoff	73	Male	Lung cancer	10 spindle-shaped cells averaging 20 microns in diameter. These cells were of uniform appearance, with numerous adherent platelets. Cytologic detail showed within the nucleus a fine reticualr distribution of chromatin, with a slight tendency to clump and an indistinct nuclear membrane. Several nuclei contained small nucleoli with a narrow, clear perinucleolar zone. The cytoplasm was moderately abundant and for the most part evenly distributed, with some vacuolization	6.7	128	226	No	No	-	No	-	-	Yes	6 days
1976	[1]	Carey et al.	57	Female	Breast cancer	Clear, deep blue to grey abundant cytoplasm, large somewhat folded nuclei and two or more prominent nucleoli. Cells resembled megaloblasts and undifferentiated histiocytes (reticulum cells)	6.15-36.1	Anemia (exact value not provided)	93-168	Yes	Yes	Acute leukemia	Yes	No	-	Yes	10 months
1976			49	Female	Breast cancer	Rare monocytoid cells with single or double prominent nucleolus and abundant pink-tinged cytoplasm	-	34	65	Yes	Yes	Acute myeloid leukemia	Yes	No	-	Yes	-
1977	[15]	Myerowitz et al.	58	Female	Breast cancer	Abnormal cells tended to occur in clusters, with a measure of 20-30 µm, and the nuclear-cytoplasmic ratio was high. The nuclei frequently contained large nucleoli and clumped chromatin. The cytoplasm was lightly basophilic and several cells showed a single intracytoplasmic vacuole. No Auer rods or cytoplasmic granules were observed	19.5	-	56	Yes	No	-	Yes	-	-	Yes	11 days
1979	[84]	Dannaher et al.	57	Male	Lung cancer	Circulating primitive cells with large round nuclei rich in reticular chromatin and agranular cytoplasm	9.8	69	23	Yes	Yes	Acute leukemia	No	-	-	Yes	3 days
1979	[90]	Ejeckam et al.	48	Female	Lung cancer	Clumped cells larger than normal lymphocytes, had hyperchromatic pleomorphic nuclei and were peroxidase-negative. Blast-like cells with large nuclei, scanty cytoplasm, fine and evenly distributed chromatin and one or two nucleoli	8.6	96	26	Yes	Yes	Acute leukemia	No	Yes	Extensive petechiae, ecchymoses, suggestive of coagulopathy	Yes	11 days
1979	[72]	Krause	26	Female	Rhabdomyosarcoma	Cells with merging blue cytoplasm and indistinct cell borders. The nuclei were generally oval and hyperchromatic with barely discernible nucleoli. The cells ranged from 10 to 20 µm in diameter)	12.3	74	-	No	No	-	No	No	-	Yes	5 days
1980	[89]	Solanki et al.	62	Male	Lung cancer	Abnormal mononuclear cells characterized by deep blue cytoplasm, multiple cytoplasmic vacuoles, an immature nucleus with prominent folding in some, prominent nucleoli, and a high nucleocytoplasmic ratio	11.9	121	20	Yes	No	-	No	No	-	Yes	2 weeks
1982	[88]	Thompson et al.	63	Female	Lung cancer	Clumping of the cells; large cell size; high nuclear-to-cytoplasmic ratio; absence of Auer rods or granules	7.3	151	44	Yes	No	-	No	Yes	Thrombocytopenia-associated bleeding (not clearly diagnosed as DIC)	Yes	5 days
1984	[14]	Gallivan and Lokich	55	Female	Melanoma	Large hyperchromatic nuclei and high nuclear cytoplasmic ratio	28	111	-	Yes	No	-	Yes	No	-	Yes	15 days
1984			80	Male	Transitional cell carcinoma	-	0.1	Anemia (exact value not provided)	22	No	No	-	Yes	Yes	Disseminated intravascular coagulation	Yes	8 weeks
1985	[99]	Irie et al.	35	Male	Germinal cell carcinoma	Abnormal cells, which have huge, bizarre and hyperchromatic nuclei, 40-50 µm, in size, and scant cytoplasm. No Auer rods or cytoplasmic granules were observed	11.2	89	71	Yes	No	-	Yes	No	-	Yes	1.5 months
1987	[75]	Yam et al.	61	Female	Breast cancer	Large, malignant mononuclear cells	1.9	60	16	Yes	Not available	Not available	Not available	Not available	Not available	Not available	Alive at time of publication
1990	[76]	Luggasy et al.	55	Female	Breast cancer	-	2.6	104	26	Yes	Not available	Not available	Not available	Not available	Not available	Not available	3 months
1990			67	Female	Breast cancer	-	1.2	72	70	Yes	Not available	Not available	Not available	Not available	Not available	Not available	5 weeks
1991	[29]	Maldonado et al.	18	Female	Rhabdomyosarcoma	Small round vacuolated primitive cells, many of which formed clumps of varying sizes	16	57	20	Yes	Not available	Not available	Not available	Not available	Not available	Not available	8.5 weeks
1991	[91]	Sabattini et al.	16	Male	Rhabdomyosarcoma	Circulating undifferentiated blasts	-	-	-	Yes	No	-	No	No	-	Yes	3 months
1991			24	Male	Rhabdomyosarcoma	Circulating undifferentiated blasts	-	-	-	Yes	No	-	No	No	-	Yes	3 months
1991			36	Male	Rhabdomyosarcoma	Circulating undifferentiated blasts	-	-	-	Yes	No	-	No	No	-	Yes	3 months
1992	[27]	Aboulafia	62	Female	Breast cancer	An increased nuclear-to-cytoplasmic ratio with hyperchromatic nuclei and measured between 18 and 22 microns in diameter	23.9	161	100	-	No	-	Yes	No	-	Yes	1 day
1995	[66]	Brace et al.	80	Female	Breast cancer	Circulating nucleated cells with tendency to cluster	34	108	114	Yes	No	-	Yes	Yes	Microangiopathic Hemolytic Anemia	Yes	Few days
1996	[16]	Nasr et al.	55	Female	Cancer of unknown primary origin	These cells shared some morphologic characteristics of hematopoietic cells but were often arranged in small clumps	8.4	125	80	Yes	No	-	No	No	-	No	Alive at time of publication
1996	[92]	Morandi et al.	18	Female	Rhabdomyosarcoma	Medium and large undifferetiated cells with faintly basophilic cytoplasm	7.2	80	29	Yes	No	-	No	No	-	No	-
1999	[87]	Sile et al.	51	Female	Lung cancer	Large mononuclear cells characterized by deep cytoplasm, cytoplasmic vacuoles, high nuclear-cytoplasmic ratio, fine nuclear chromatin, and indistinct nucleoli	13.7	120	276	-	No	-	Yes	No	-	Yes	2 days
1999	[93]	Trefzer et al.	48	Male	Melanoma	Atypical plasmacytoid, large pleomorphic cells, with abundant cytoplasm, eccentric nuclei, and marked anisokaryosis	22.5	Moderate anemia (exact value not provided)	Thrombocytopenia (exact value not provided)	Yes	Yes	Acute leukemia	Yes	No	-	Yes	9 days
2000	[77]	Rodríguez-Salas et al.	65	Female	Breast cancer	Large size, one nucleus with finely dispersed chromatin, basophilic cytoplasm without granulation and imprecise borders	12.3	90	128	Yes	No	-	Yes	No	-	Yes	2 weeks
2001	[19]	Séronie-Vivien et al.	60	Female	Breast cancer	Blastic lymphoid-like cells with high nucleus/cytoplasm ratio light blue to grey cytoplasm, irregular limits, fine vacuoles, weak chromatin condensation, frequent nucleoli	23.8	108	384	Yes	Yes	Lymphoma or acute leukemia	Yes	Yes	Multiple deep venous thromboses	Yes	3 months
2003	[86]	Bedolla and Stemmerman	61	Male	Lung cancer	-	Leukopenia (exact value not provided)	Anemia (exact value not provided)	Thrombocytopenia (exact value not provided)	Yes	Yes	Lymphoid malignancy	No	No	-	Yes	13 days

Supplemental Table 1. (continued)

Year of publication	Ref	Authors	Age	Gender	Cancer type	CTC features	Leucocytes ($x10^9/L$)	Hemoglobin (g/L)	Platelets ($x10^9/L$)	BM infiltration	Misdiagnosing with malignant hematologic diseases?	Which disease?	Personal history of cancer	Coagulation complications?	Which coagulation complications?	Death	Time to death
2004	[69]	Chen et al.	13	Male	Rhabdomyosarcoma	Mononuclear 'blasts' with eccentric nuclei and occasional cytoplasmic vacuolation	6.5	116	<10	Yes	Yes	-	No	Yes	Petechiae, ecchymoses	No	Alive at time of publication
2005	[94]	Tam et al.	66	Male	Merkel cell carcinoma	-	Leukopenia (exact value not provided)	Anemia (exact value not provided)	Thrombocytopenia (exact value not provided)	Yes	No	-	Yes	No	-	Yes	5 days
2005			55	Male	Merkel cell carcinoma	-	Leukopenia (exact value not provided)	Anemia (exact value not provided)	Thrombocytopenia (exact value not provided)	Yes	No	-	Yes	No	-	No	Was lost to follow-up at 6 weeks
2006	[20]	Pitini et al.	38	Female	Breast cancer	Blastic lymphoid-like cells with a high nucleocytoplasmic ratio, a light blue to grey cytoplasm with irregular limits at times, weak chromatin condensation, and frequent presence of nucleoli	1.2	71	40	Yes	No	-	No	No	-	No	Alive at time of publication
2006	[24]	Marrinucci et al.	38	Female	Breast cancer	CTCs exhibit a high degree of pleomorphism including CTCs with high and low nuclear-to-cytoplasmic ratios along with CTCs exhibiting early and late apoptotic changes	-	-	-	Yes	No	-	Yes	No	-	Yes	3 months
2008	[5]	Chang et al.	48	Female	Breast cancer	Large abnormal cells with high nucleus/cytoplasm ratio, large nucleolus, basophilic cytoplasm, and intracytoplasmic vacuoles	28.2	71	57	Yes	Yes	Acute lymphocytic leukemia	Yes	Yes	Subdural hemorrhage due to thrombocytopenia	Yes	9 months
2008	[96]	Misawa et al.	51	Male	Colorectal cancer	Nonhematopoietic cells in peripheral blood appear in lose clusters	25.2	-	12.9	Yes	No	-	Yes	Yes	Disseminated intravascular coagulation	Yes	3 days
2009	[25]	Tran et al.	67	Male	Melanoma	Large, pleomorphic, noncohesive tumor cells. These cells contained mildly irregular nuclei and abundant basophilic cytoplasm with variable vacuolization. Some cells contained melanin pigments	1.8	96	49	Yes	No	-	Yes	No	-	Yes	1 week
2009	[26]	Usnarska-Zubkiewicz et al.	31	Male	Pancreatic cancer	Atypical immature pleomorphic cells with navy blue cytoplasm and osteoclast-like multinuclear giant cells	15.6	74	56	Yes	Yes	Acute leukemia	No	No	-	Yes	3 weeks
2010	[95]	Hartley et al.	71	Male	Merkel cell carcinoma	Abnormal carcinoma-like cells	Leukopenia (exact value not provided)	Anemia (exact value not provided)	Thrombocytopenia (exact value not provided)	Yes	No	-	Yes	Yes	Deep venous thrombosis	Yes	2 months
2010	[30]	Jelić-Puskarić et al.	14	Female	Rhabdomyosarcoma	Primitive, immature, medium-sized cells. The cells were mostly single, partly in small clusters and 'rosettes', with reticular chromatin structure, and moderate to abundant, frequently finely vacuolated and in part elongated cytoplasm	28.5	85	15	Yes	Yes	Acute leukemia	No	No	-	No	-
2011	[78]	Robier et al.	66	Female	Breast cancer	Large nuclei with one or more prominent nucleoli and slightly gray to basophilic cytoplasm; tended to occur in clusters of up to four	18.5	88	109	Yes	No	-	Yes	Yes	Disseminated Intravascular Coagulation and Thrombotic Microangiopathy	Yes	3 days
2013	[47]	Milosevic et al.	18	Female	Rhabdomyosarcoma	Atypical, with heterogeneous morphology, resembling lymphoblasts, myeloblasts, and even erythroblasts… round, oval or spindle-shaped, with round, oval or irregular-shaped nuclei, light chromatin pattern, inconspicuous nucleoli, and a scant cytoplasm	5.34	85	32	Yes	Yes	Acute lymphocytic leukemia	No	No	-	No	-
2014	[97]	van Bunderen et al.	65	Female	Colorectal cancer	A sporadic atypical cell of unknown origin, resembling a seal ring	14.1	98	136	Yes	No	-	No	Yes	Disseminated intravascular coagulation	Yes	8 months
2015	[49]	Ogura et al.	65	Female	Breast cancer	Noncohesive and had round nuclei and cytoplasm with microvilli resembling lymphocytes or plasma cells in form	22.2	137	315	Yes	Yes	Lymphoma or plasma cell leukemia	No	No	-	No	Alive at time of publication
2017	[31]	Johnsrud and Pina-Oviedo	33	Female	Breast cancer	Large atypical cells (~50 µm) with vacuolated cytoplasm resembling myeloid/erythroblasts or Burkitt lymphoma cells	Leukoerythroblastosis (exact value not provided)	-	Thrombocytopenia (exact value not provided)	-	No	-	Yes	Yes	Disseminated intravascular coagulation	Yes	Shortly after CTC detection (exact interval not specified)
2017	[48]	Jain et al.	50	Male	Breast cancer	Atypical cells with high nuclear/cytoplasmic ratio. The nuclei showed relatively clumped chromatin with moderate amount of lightly basophilic cytoplasm.	12.5	66	17	Yes	Yes	Acute leukemia	Yes	-	-	No	Alive at time of publication
2018	[6]	Ronen et al.	41	Female	Breast cancer	Rare atypical mononuclear cells. Large, with round nuclei, dispersed chromatin, and abundant pale basophilic cytoplasm	7.8	36	7	-	No	-	Yes	Yes	Microangiopathic Hemolytic Anemia	Yes	1 month
2018			54	Female	Lung cancer	Rare large atypical cells. The cells showed irregular nuclei, prominent nucleoli, and basophilic, abundantly vacuolated cytoplasm	6.4	72	117	Yes	No	-	No	-	Microangiopathic Hemolytic Anemia	No	Alive at time of publication
2018			72	Male	Prostate cancer	Clusters of atypical cells. The large cells showed prominent nucleoli and moderately abundant basophilic cytoplasm	7.1	82	110	-	No	-	Yes	No	-	Yes	2 months
2018			58	Female	Cancer of unknown primary origin	Atypical cells with round, regular nuclei, dispersed to reticular chromatin, small nucleoli, and abundant, pale basophilic, vacuolated cytoplasm	10.1	85	416	Yes	No	-	Yes	Yes	Deep vein thromboses	No	Was lost to follow-up at 4 months
2018			57	Female	Breast cancer	Cells with the same features as described in the aspirate smear (large cells with regular nuclei, coarsely reticular chromatin, prominent nucleoili, and abundant vacuolated cytoplasm)	5.6	80	14	Yes	No	-	Yes	No	-	Yes	26 days
2018			69	Male	Lung cancer	Rare single cells with large size, large, folded nuclei, distinct nucleoli, and scanty basophilic cytoplasm	13.6	112	431	-	No	-	Yes	No	-	No	Alive at time of publication
2018	[6] | [79]	Ronen et al. / Rowan et al.	54	Female	Breast cancer	Large size with round nuclei, coarse chromatin, inconspicuous nucleoli, and moderately abundant, dense, basophliic cytoplasm	16.7	114	184	Yes	No	-	Yes	No	-	Yes	8 days
2018	[80]	Fratamico et al.	76	Female	Breast cancer	Large with large immature nuclei, prominent nucleoli, and a moderate amount of cytoplasm. Cells that resembled promyelocytes with Auer rods frequently seen in acute promyelocytic leukemia.	18.2	-	-	Yes	Yes	Acute promyelocytic leukemia	No	Yes	Coagulopathy with altered INR, PT, APTT and D-dimer	No	Alive at time of publication
2018	[85]	Sandberg and der Bakker	63	Male	Lung cancer	Atypical blast-like cells with large nuclei and a scant basophilic cytoplasm	Leukocytosis with neutrophil left shift (exact value not provided)	Anemia (exact value not provided)	Thrombocytopenia (exact value not provided)	Yes	No	-	No	-	-	Yes	8 days

Supplemental Table 1. (continued)

Year of publication	Ref	Authors	Age	Gender	Cancer type	CTC features	Leucocytes ($x10^9$/L)	Hemoglobin (g/L)	Platelets ($x10^9$/L)	BM infiltration	Misdiagnosing with malignant hematologic diseases?	Which disease?	Personal history of cancer	Coagulation complications?	Which coagulation complications?	Death	Time to death
2019	[81]	Oliver-Caldes et al.	65	Female	Breast cancer	Abnormal cells morphologically similar to those seen in the marrow a year previously	Leukoerythroblastosis (exact value not provided)	67	42	Yes	No	-	Yes	-	-	No	Alive at time of publication
2019	[67]	Samal et al.	60	Male	Lung cancer	-	11	82	210	-	No	-	Yes	-	-	Yes	Few hours
2020	[33]	Rakislova et al.	32	Male	Gastric cancer	Morphologically malignant epithelial-like cells showing big size and prominent nucleoli. Ocassionally, large signet ring epithelial cell	7	68	39	Yes	No	-	No	Yes	Coagulopathy with altered PT	No	-
2020	[50]	Barksdale and Leith	76	Female	Breast cancer	Large abnormal cells with eccentric nuclei, cup-shaped nuclear invaginations and variable blue-gray cytoplasm	13	72	77	Yes	Yes	Plasma cell leukemia	No	-	-	No	-
2021	[17]	Jain and Wang	60	Male	Prostate cancer	Atypical cells with irregular nuclear contour	4.2	84	55	Yes	No	-	Yes	-	-	No	Alive at time of publication
2022	[70]	Roque García et al.	71	Female	Breast cancer	Large cells with abundant basophilic agranular cytoplasm, eccentric nucleus with fine chromatin, and visible nucleoli, resembling plasma cells	15.8	77	150	Yes	Yes	Multiple myeloma	No	-	-	Yes	3 months
2022	[82]	Vila et al.	73	Female	Breast cancer	Several neoplastic cells with round nuclei, finely dispersed chromatin, small nucleoli, and basophilic cytoplasm sometimes with hairy edges	12	121	206	Yes	Yes	Hairy cell leukemia	Yes	Yes	Retinal vein thrombosis	Yes	Few weeks
2022	[51]	Pathak et al.	67	Female	Breast cancer	Atypical cells having blastic/plasmablastic morphology. Focal loose clustering of these cells was also noted	Mild neutrophilia (exact value not provided)	Mild normocytic anemia (exact value not provided)	Normal	Yes	Yes	Plasma cell leukemia	Yes	Yes	Microangiopathy	No	-
2022	[28]	Tariq	57	Female	Breast cancer	Large bizarre cells with irregular nuclear contours and multiple prominent nucleoli. Clusters of abnormal cells	-	88	12	Yes	No	-	Yes	Yes	Microangiopathic Hemolytic Anemia	Yes	6 days
2023	[71]	Trinder et al.	31	Female	Breast cancer	Large atypical cells	Leukoerythroblastosis (exact value not provided)	99	10	Yes	Yes	Thrombotic thrombocytopenic purpura	No	Yes	Microangiopathic Hemolytic Anemia	Yes	A short time
2023	[21]	Vanrenterghem et al.	45	Female	Breast cancer	3–4 RBC in size, with a round to oval nucleus, a moderate N/C ratio, mature reticular chromatin with multiple dark nucleoli and moderate basophilic, often vacuolized, frayed cytoplasm	-	70	69	Yes	Yes	Thrombotic thrombocytopenic purpura	Yes	Yes	Microangiopathic Hemolytic Anemia	Yes	Few days
2023	[22]	Zhang et al.	12	Male	Ewing sarcoma	Large abnormal cells dispersed on the center smear, with a high nucleocytoplasmic ratio, round nucleus, scant blue cytoplasm, and fine chromatin, resembling lymphoblast-like morphology	11.31	138	32	-	No	-	Yes	-	-	No	-
2023	[100]	Su et al.	Middle-aged	Female	Clear cell sarcoma	Large amounts of malignant cell clusters accompanied with some pigment particles. Some abnormal cells displayed with round eccentric nuclei, one to several prominent nucleoli and abundant cytoplasm containing multiple microvacuoles, mimicking plasmablastic morphology	-	-	-	Yes	No	-	Yes	-	-	No	-
2023	[32]	Farkh et al.	80	Female	Breast cancer	Discohesive, large, atypical cells with abundant basophilic vacuolated cytoplasm, irregular pseudolobed nuclei, with weak chromatin condensation and blue prominent nucleoli	13.5	130	143	-	No	-	Yes	Yes	Disseminated intravascular coagulation	Yes	A short time
2024	[83]	Tassin et al.	70	Female	Breast cancer	Rare, atypical mononuclear cells with cytoplasmic vacuolation	10.4	96	77	Yes	No	-	Yes	-	-	Yes	2 months
2024	[35]	Briers et al.	56	Female	Breast cancer	Atypical cells, mainly located at the feathered edge	-	-	-	-	-	-	No	Yes	Disseminated intravascular coagulation	Yes	4 days
2024	[36]	Wood et al.	73	Female	Breast cancer	-	Mild neutropenia (exact value not provided)	-	-	-	-	-	Yes	-	-	No	Alive at time of publication
2024	[7]	Suner et al.	79	Female	Endometrial cancer	Large with round nuclei, dispersed chromatin, prominent nucleoli and abundant basophilic cytoplasm	11.2 (7.9-16.3)	103 (88-118)	103 (62-193)	-	-	-	Yes (16/22)	Yes	Increased D-dimer, thrombosis (4/22)	Yes	0.9 months (0.25-5)
2024			30	Female	Breast cancer	Medium to large cells with round nuclei, dispersed chromatin, small nucleoli and abundant vacuolated cytoplasm. Two cases had atypical features: one with signet ring-like cells and another with granulated tumor cells											
2024			34	Female	Breast cancer												
2024			50	Female	Breast cancer												
2024			53	Female	Breast cancer												
2024			62	Female	Breast cancer												
2024			68	Female	Breast cancer												
2024			69	Female	Breast cancer												
2024			71	Female	Breast cancer												
2024			75	Female	Breast cancer												
2024			76	Female	Breast cancer												
2024			76	Female	Breast cancer												
2024			77	Female	Breast cancer												
2024			80	Female	Breast cancer												
2024			82	Female	Breast cancer												
2024			82	Female	Breast cancer												
2024			86	Female	Breast cancer												
2024			55	Male	Lung cancer	Medium to large cells with irregular nuclei, inconspicuous nucleoli and scanty basophilic cytoplasm											
2024			66	Male	Lung cancer												
2024			75	Female	Lung cancer												
2024			79	Female	Lung cancer												
2024			63	Male	Biliary duct cancer	Very atypical, with medium to very large cells, round nuclei, sometimes multinucleated, reticulated to coarse chromatin, prominent nucleoli and abundant basophilic cytoplasm											

Supplemental Table 1. (continued)

Year of publication	Ref	Authors	Age	Gender	Cancer type	CTC features	Leucocytes (x10^9/L)	Hemoglobin (g/L)	Platelets (x10^9/L)	BM infiltration	Misdiagnosing with malignant hematologic diseases?	Which disease?	Personal history of cancer	Coagulation complications?	Which coagulation complications?	Death	Time to death
2024	[23]	Singh et al.	44	Female	Breast cancer	Atypical large mononuclear cells, with high nucleocytoplasmic ratio, a prominent nucleolus, coarse chromatin, and moderate amount of ill-defined pale basophilic cytoplasm.	1.5	82	30	Yes	Yes	Acute leukemia	Yes	Yes	Spontaneous bruising	Yes	15 days
2024	[34]	Rullo et al.	65	Male	Bladder cancer	CTCs exhibiting signet ring morphology (CTCs measured using CellSearch® system)	-	38	34	Yes	No	-	No	Yes	Pulmonary embolism	Yes	2 months
